# Effect of data harmonization of multicentric dataset in ASD/TD classification

**DOI:** 10.1186/s40708-023-00210-x

**Published:** 2023-11-25

**Authors:** Giacomo Serra, Francesca Mainas, Bruno Golosio, Alessandra Retico, Piernicola Oliva

**Affiliations:** 1 Department of Physics, University of Cagliari, Cagliari, Italy; 2 National Institute for Nuclear Physics (INFN), Cagliari Division, Cagliari, Italy; 3 National Institute for Nuclear Physics (INFN), Pisa Division, Pisa, Italy; 4 Department of Chemical, Physical, Mathematical and Natural Sciences, University of Sassari, Sassari, Italy

**Keywords:** ABIDE, Multi-site data, Harmonization, Machine learning, Autism spectrum disorder

## Abstract

Machine Learning (ML) is nowadays an essential tool in the analysis of Magnetic Resonance Imaging (MRI) data, in particular in the identification of brain correlates in neurological and neurodevelopmental disorders. ML requires datasets of appropriate size for training, which in neuroimaging are typically obtained collecting data from multiple acquisition centers. However, analyzing large multicentric datasets can introduce bias due to differences between acquisition centers. ComBat harmonization is commonly used to address batch effects, but it can lead to data leakage when the entire dataset is used to estimate model parameters. In this study, structural and functional MRI data from the Autism Brain Imaging Data Exchange (ABIDE) collection were used to classify subjects with Autism Spectrum Disorders (ASD) compared to Typical Developing controls (TD). We compared the classical approach (external harmonization) in which harmonization is performed before train/test split, with an harmonization calculated only on the train set (internal harmonization), and with the dataset with no harmonization. The results showed that harmonization using the whole dataset achieved higher discrimination performance, while non-harmonized data and harmonization using only the train set showed similar results, for both structural and connectivity features. We also showed that the higher performances of the external harmonization are not due to larger size of the sample for the estimation of the model and hence these improved performance with the entire dataset may be ascribed to data leakage. In order to prevent this leakage, it is recommended to define the harmonization model solely using the train set.

**Supplementary Information:**

The online version contains supplementary material available at 10.1186/s40708-023-00210-x.

## Introduction

Autism Spectrum Disorders (ASD) are a neurodevelopmental behavioral disorder [1, 2] and refer to a broad range of conditions manifesting as deficits in social communication and interaction such as reduced sociability or empathy, repetitive behavior. From a neurological perspective, numerous investigations have been done on the potential link between ASD and variation in the structure and function of different brain regions. Structural studies usually focus on volumetric and morphometric analyses to examine differences in brain anatomy. It has been studied how ASD could alter the symmetry between the two hemispheres [3] of the brain. In children it has been reported an increase in total brain volume as well as an enlargement of the left superior temporal gyrus. However this trend is not well defined for older ages [4]. Functional neuroimaging research mainly focuses on impaired connectivity in resting-state images. Different studies pointed out a reduced information processing due to synaptic dysfunction that manifests in a reduced or altered brain functional connectivity [5, 6].

Neuroscience data are frequently made of a large number of features (hundreds or thousands), each of them often containing only a small amount of information. Moreover, the differences between classes are small and consequently hard to detect (low effect size). Machine learning is one of the most promising tools to deal with complex, non-linear problems and is often used in the analysis of neuroimaging data, in particular in the identification of the most discriminative features between different classes of subjects. A drawback of machine learning is that datasets of large dimension are required, in order to obtain a model able to gention and to maintain the same level of performance both on training and on new data.

Unfortunately, the number of subjects in these datasets is often small (on the order of tens), since it is limited by the number of subjects that can reasonably be involved in a study (low sample size). The reduced number of subjects implies a serious risk of data overfitting, with a consequent need for data reduction methods and accurate cross-validation schemes. However, data reduction may potentially undermine the detectability of a low effect size. Jamalabadi et al. [7] show that cross-validation in low sample size/low effect size (LSS-LES) data possesses some counterintuitive properties. In particular, classification rates below the chance are often observed in LSS-LES data. But also higher performances may not necessarily represent larger differences between classes.

Moreover, the dependence of neuroimaging features on covariates as age, sex and full intelligence quotient (FIQ) has to be taken into account. Age, for example, affects both structural and functional data, with an observed overgrowth of the brain volume and hyper-connectivity in toddlers and subsequent decrease with increasing age [8]. Moreover, some brain areas exhibit an increased functional connectivity in female subjects, and some brain structures show a different aging effect between male and female subjects [9]. Also the eye status at scan affects the functional connectivity, with strong differences and higher connectivity in different brain areas between subjects with open or closed eyes [10]. Furthermore, patients with closed eyes may fall asleep during MRI scan, with consequent heavy modification in the functional brain activity.

In machine-learning-based analyses, the increase in the number of subjects in the dataset makes the classifier less prone to overfitting. Consequently, a better understanding of the relationship between features and class labels is possible, maximizing the classifier ability to distinguish between the two groups. Given the reduced size of typical neuroimaging datasets, the scientific community is moving towards the realization of multicentric datasets. ABIDE (Autism Brain Imaging Data Exchange) is a project aimed at collecting and sharing structural and functional magnetic resonance images, together with phenotypic data, of individuals with autism spectrum disorder and typically developing (TD) controls. This data sample has been made publicly available in two successive collections: ABIDE I [11] and ABIDE II [12]. ABIDE I involves 17 international labs and contains 1112 patients, of which 539 are subjects with ASD and 573 are TD. ABIDE II involves 19 international labs and 1114 patients, divided into 521 subjects with ASD and 593 TD controls. Despite the great advantage of a larger sample size, the difficulty of the analysis on a multicentric dataset is related to the need to eliminate the heterogeneity of the data caused by the different acquisition protocols or scanners of the individual sites. Ignoring this heterogeneity could have a significant impact on the results.

A typical approach to deal with this heterogeneity is the harmonization of multicentric datasets, which is used to overcome several challenges such as differences in data collection protocols, data formats, and patient populations. A recent review paper [13] offers an extensive analysis of the various statistical and deep learning techniques that have been developed for image harmonization.

In particular, Fortin et al. [14] propose a data harmonization protocol that aims to eliminate the site effect in neuroimaging, while preserving dependence of the features on biologically significant covariates. This protocol is an adaptation of the ComBat method developed by Johnson et al [15] to remove the batch effect in genomic data. The NeuroHarmonize tool, proposed by Pomponio et al. [16], is suitable to harmonize pooled datasets in the presence of non-linear covariate trends. The harmonization model parameters are calculated from the TD population. The harmonization transformation is then applied to the whole dataset. The underlying assumption of the NeuroHarmonize method is that all measurements in a given sample are drawn from the same reference distribution, even though there may be differences in age, gender, and other variables among the subjects within each sample. Patients with an altered functional brain map could violate this assumption, therefore their inclusion in the modeling phase may attenuate the differences between controls and cases attributable to the disorder [16]. This approach is extensively used in harmonization of multicentric datasets in the field of MRI imaging [17–20]. The method is generally applied as a preprocessing step of the whole dataset, before the (cross-validated) classification. However, the use of the whole dataset to estimate the parameters of the harmonization model, in principle introduces a data leakage, since also the subjects in the test set are used in this estimation. T. Li et al. [21] observed that the separation of harmonization from downstream analyses leads to an artificial correlation between originally-independent subjects. This occurs because batch effects are estimated using all subjects in the dataset. Consequently, each harmonized data point is influenced by all other data points in the dataset, thus leading to correlations between them. This induced correlation, if not accounted for, could result in downstream analyses yielding exaggerated or reduced findings. A more rigorous approach requires estimating the parameters of the harmonization model only on the train set, leaving the classification model completely blind to the test set. On the other hand, this second approach may result in less-accurate estimation of the harmonization parameters and hence in reduced performances.

In this work, we investigated different approaches to the harmonization of a multicentric dataset and show the differences in site distinguishability, classification performances and most important features involved in the classification.

## Materials and methods

### Feature generation

Structural images were processed with Freesurfer [22] 6.0 with the recon-all pipeline2. Among the all features generated by the Freesurfer processing pipeline, as in Saponaro et al. [23], the following brain measures were selected:

- volume, mean and standard deviation of the thickness of 62 structures (31 per hemisphere) from the Desikan–Killiany–Tourville Atlas [24], for a total of 186 cortical features;
- 26 volumes of subcortical structures and corpus callosum;
- 9 global quantities: mean thicknesses, cortex volumes, cerebral white matter volumes, for both left and right hemispheres; cerebrospinal fluid volume, total gray volume and the volume of segmented brain without ventricles;

In this way, a total number of 221 brain structural features were obtained.

Connectivity data were obtained using C-PAC, which is a configurable, open-source pipeline, based on the Nipype platform, to perform motion correction, slice timing correction, band-pass filtering, spatial smoothing and registration.

After preprocessing, average time series were extracted using the Harvard-Oxford parcellation [25], obtaining 110 timeseries for each subject. Out of these 110 ROIs, 7 were removed because of null time series in a significant number of patients. The result is a total of 103 ROIs actually used per each patient.

The Pearson correlation between two time series was used to measure functional connectivity. The Pearson correlation coefficients were Fisher z-transformed, in order to make them approximately normally-distributed [26].

The correlation was computed for each pair of brain areas, resulting in 5253 ($N_{comb} = \frac{1}{2} n(n-1)$ independent combination for n=103 time series) connectivity feature for each subject.

### Data selection

In order to cope with the heterogeneity of the dataset, we applied several selection criteria, based on phenotypic data (gender, eye status) and data quality, in order to make the dataset as homogeneous as possible.

Since data selection obviously reduces the amount of available data, we applied different selection strategies, with an increasingly restrictive approach.

- As a first approach we started from the dataset used in Saponaro et al. [23]: males, age between 6 and 40 years. From the initial set of 2226 subjects of the ABIDE collection, we excluded 65 subjects due to unsuccessful preprocessing with Freesurfer and 262 subjects due to unsuccessful C-PAC preprocessing. Thus, we obtained 1899 subjects processed with both structural and functional pipelines. From this dataset, we selected only male subjects aged between 6 and 40 years, excluding 1211 subjects. According to these selection criteria, the resulting dataset consists of 688 subjects. This dataset will be referred to as minSC (minimal selection criteria).
- A second approach was driven by quality controls in fMRI data. We limited our analysis to males, eyes-open, for which Full Intelligent Quotient is available (FIQ>0). Moreover, we used the Mean Framewise Displacement (*mean fd* parameter provided by CPAC) to assess the movement of the patient’s head from one volume to the next one. *Mean fd* is estimated by summing the absolute displacement values calculated at each timepoint. Subjects with *mean fd* values less than 3 MAD (Median Absolute Deviation) were regarded as outliers. Furthermore the patients excluded from minSC, due to a failure in the preprocessing pipelines, are excluded also from this set. This dataset will be referred to as fQC (selection criteria based on fMRI quality controls) and contains 618 subjects.
- In order to reduce the impact of covariates on classification, we also limited our analysis to a narrower age range (9–20 years). The age selection is applied post-harmonization, allowing the harmonization procedure to estimate the covariate dependence on a wider age range. Due to the reduction of subjects, we also removed from the analysis sites with less than 30 subjects. For these additional selection criteria, the label as (age selection) is added to the dataset name. These datasets consist of 520 and 473 subjects, respectively, for minSCas and fQCas.

For each selection, the database numerosity is reported in Table 1, while the numerosity for each site and for each dataset, is reported in Additional file 2: Table S1, with the complete list of subjects IDs for each selection, for reproducibility purpose.

**Table 1:** Numerosity of the different selected datasets

	minSC	minSCas	fQC	fQCas
TD	344	265	320	247
ASD	344	255	298	226
Total	688	520	618	473

The total number of subjects, the total number of TD and of ASD are reported for each dataset

*minSC* minimal selection criteria, * minSCas* minimal selection criteria age selection, * fQC* fMRI quality controls, *fQCas* fMRI quality controls age selection

### Classification strategy

The supervised binary classification of ASD and TD classes was performed using Support Vector Machines (SVM) [27]. SVMs are particularly effective at handling noisy and correlated features and can yield superior results compared to other classifiers when dealing with datasets that have a small number of samples and a large number of features [28]. We used the SVM implemented in the Scikit-learn [29], a Python open-source machine learning library, in its variant with the Gaussian radial basis function (RBF) kernel, using the default parameters. We employed a stratified 5-fold cross-validation scheme to train the Support Vector Classifier (SVC) model, with the objective of achieving balanced training across the classes and across the sites. This scheme was repeated 50 times to ensure robustness of the results. We applied a feature scaling function, the Scikit-learn RobustScaler, which performs a median subtraction and scales the data using the interquartile range (IQR). The feature scaling function was computed within each fold of the 5-fold cross-validation scheme and for each repetition.

The classification performance was evaluated using the area under the ROC curve (AUC). The receiver operating characteristic (ROC) curve [30] represents the performance of the classifier at different decision thresholds and the AUC is a global index that can be used to compare the ROC curves of different classifiers. The AUC represents the probability of correctly ranking a case–control pair and can be estimated from the ROC curve [31]. The AUC was calculated for each fold and repetition, and the results were aggregated across the 5 test folds and 50 repetitions to obtain the mean and standard deviation of the AUC, which served as performance metrics for the SVC model.

### Harmonization approaches

We used the NeuroHarmonize [16] package harmonization tool. The model is always estimated using the TD subjects, and then applied to all subjects. The utilization of the harmonization process in case–control comparative studies necessitates having access to suitable control population data. Essentially, the harmonization model parameters are derived from the TD population, and subsequently, the harmonization transformation is applied to all the subjects. The underlying premise of the NeuroHarmonize approach is that each measurement in the samples originates from the same reference distribution, despite potential variations in age, gender, and brain features among subjects in each sample. The inclusion of patients with structural or functional brain alterations could challenge this assumption and, moreover, incorporating them into the harmonization process would dampen disease-related effects [16]. We used the age and site as covariates, supposing a linear dependence on age. ASD status is not used as covariate, since it has to be supposed unknown for the test set.

We implemented the following 4 different harmonization strategies:

- No harmonization. Data is used without any attempt to correct potential site effects.
- External harmonization. The harmonization is applied before the CV scheme, as a preprocessing step, using all the TD of the dataset to estimate the parameters of the harmonization model. Hence the harmonization is outside the CV loop.
- External harmonization, by using 4/5 of the TD subjects. The harmonization is applied before the CV scheme, using 4/5 of the TD of the dataset to estimate the parameters of the model. This approach wants to replicate the same numerosity of subjects in estimating the harmonization parameters of the next approach (internal harmonization), considering that we are using a 5-fold CV scheme, but still allowing data leakage in the harmonization step. At each repetition, the 4/5 of the controls are extracted randomly per each site and then assembled, in order to properly represent the numerosity of each site.
- Internal harmonization. In this approach the harmonization model is estimated by using only the TD subjects in the training set, for each fold and for each repetition of the CV scheme. Following this approach, no data leakage is possible from the test set. In this case, the harmonization is inside the CV loop.

### Efficacy of different harmonization strategies

The results of the different harmonization approaches were evaluated by testing the performance of a classifier in recognizing a site in a two-class classification problem, for each pair of sites. For the task, we used only the TD subjects of each site and the site name as a label. An SVM rbf was used as classification model, used in a k-fold (k=5) CV scheme, repeated 10 times. In order to reduce the influence of overfitting, we used the Principal Component Analysis (PCA) [32] as a dimensionality reduction method. PCA is an unsupervised machine learning technique aimed at reducing the dimension of the samples that compose the dataset. This is achieved through a linear transformation of the features: the original features are projected into a new Cartesian coordinate system. In this coordinate system, the new features, now called *Principal Components* (PCs), are ordered by variance. As a result, the PC with the highest variance is projected onto the first axis, the second PC onto the second axis, and so forth. By selecting the number of PCs to consider, the dimensionality of the problem is reduced.

In order to limit LSS-LES effects, we used the first 20 Principal Components (PCs) of the feature set and we reduced the test to the sites with at least 20 TD subjects per site. The results are reported in terms of AUC for each pair of sites. As a single metric for each approach, we also reported the median (and interquartile range) of all the AUCs.

In order to show the effect of covariate dependence preservation, we also reported the age dependence of a selected set of features, for both structural and connectivity data.

### Feature importance

One of the most relevant topics in ML analysis of neurological and neurodevelopmental disorders, is to point out the most relevant features involved in the identification of the pathology. If these results are strongly affected by the harmonization procedure, any possible finding has to be accurately investigated.

In order to quantify the contribution of each feature to the SVM model, feature permutation importance has been implemented [33]. The main idea behind permutation importance is to look at the impact of each feature on the performance of the model by measuring the decrease of a certain classification score when a feature is not available. The score variation obtained through permutation importance quantifies the importance of each feature and higher the score, higher the variation of the model performance. As a general rule one could remove the features and re-train the classifier. However this approach could be computationally complex because it needs to train the classifier a number of times equal to the number of features. To avoid re-training the classifier many times, a feature is removed from the test part of the dataset and replaced with random noise so that the feature column is still there but it does not contain useful information anymore. The noise has to be drawn from the same distribution as original feature values, otherwise the estimator might fail. The simplest way to generate this kind of noise is to shuffle values for a feature, for example using other feature values. We used the feature permutation importance implemented in the ELI5 python library [34]. The function provided in the library takes into account a trained estimator, a validation dataset, a scoring metric and it returns the importance score for each feature. The importance score indicates the resulting drop in model performance: the larger the drop in performance when a feature is shuffled, the more important that feature will be considered. We used the AUC as a scoring metric and computed the permutation importance for each fold of the 5-fold cross-validation, repeated 50 times. The final results were obtained as the average importance score across the 5 folds and 50 repetitions.

We carried out the feature permutation importance analysis for the three different harmonization approaches used in this work: no harmonization, external and internal harmonization. The purpose of the analysis was to investigate whether the most important features for the classification differ when using one approach instead of another. Through comparing the feature permutation importance scores for the three approaches, we aimed to obtain insights into whether data harmonization might affect the model’s predictive performance.

## Results

### Effects of the harmonization strategies

**Fig. 1:**
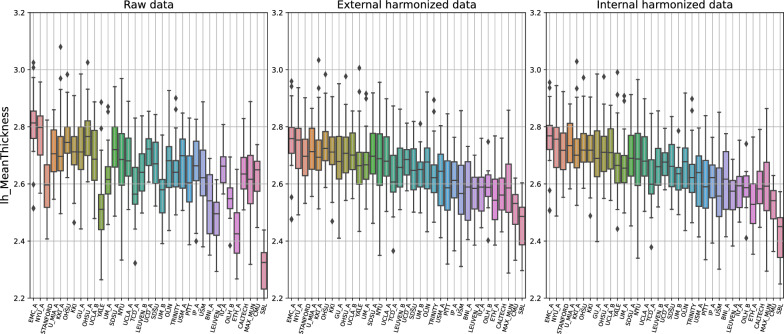
Effect of external (center) and internal (right) harmonization approaches on a structural features, left hemisphere cortical thickness, compared with the non-harmonized (left) scenario. The boxplots display the distributions of the features, grouped by site, which are sorted by increasing median age

**Fig. 2:**
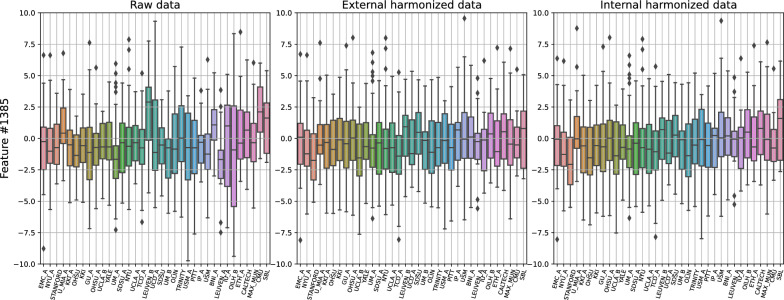
Effect of external (center) and internal (right) harmonization approaches on a connectivity features, 1385, compared with the not harmonized (left) scenario. The boxplots display the distributions of the features, grouped by site, which are sorted by increasing median age

As a first result of the different harmonization approaches, we report their effect on the age dependence of a set of selected features. Figure 1 shows the impact of external and internal harmonization techniques, compared to non-harmonized data, on the cortical thickness of the left hemisphere. The boxplots show the distributions of the features, which are grouped by site. Sites are reported in ascending order of median age of their population. The large inhomogeneity of non-harmonized data (right) almost masks the typical age dependence of these features. Harmonized data (with both external and internal harmonization) present a reduced inhomogeneity, which allows the emergence of dependence on age. Figure 2 shows the same results for feature 1385 relative to the connectivity between right postcentral gyrus and right occipital fusiform gyrus. In this case, a particular age dependence of the feature is not expected. However, the inhomogeneity of non-harmonized data is again greatly reduced by the application of harmonization technique.

**Table 2:** Median, 25 interquartile and 75 interquartile AUC performance of SVM rbf classifier in discriminating TD subjects of different sites, for each harmonization approach and each dataset, using 20 PCs

	Structural features		Connectivity features	
	minSCas	fQCas	minSCas	fQCas
No harmonization	0.99 [0.97; 1.00]	0.99 [0.96; 1.00]	0.98 [0.93; 1.00]	0.99 [0.95; 1.00]
External	0.44 [0.35; 0.50]	0.41 [0.34; 0.48]	0.53 [0.43; 0.54]	0.49 [0.45; 0.53]
4/5 External	0.50 [0.44; 0.58]	0.47 [0.39; 0.54]	0.58 [0.51; 0.60]	0.52 [0.47; 0.57]
Internal	0.67 [0.59; 0.70]	0.63 [0.61; 0.69]	0.68 [0.58; 0.69]	0.66 [0.60; 0.70]

*minSC* minimal selection criteria, * minSCas* minimal selection criteria age selection, *fQC* fMRI quality controls, * fQCas* fMRI quality controls age selection

The results of site identification in a two-class classification approach are reported in Table 2. Results are reported in terms of median, 25 interquartile and 75 interquartile of the AUC performances of SVM rbf classifiers for each pair of sites. We observe that in the first row, which refers to non-harmonized datasets, the classifier is able to perfectly identify the sites, showing a median AUC between 0.98 and 0.99, regardless of the dataset and the type of feature used. Looking at the classification performance related to external harmonization, external and 4/5 external, the median AUC values range between 0.41 and 0.58. These values are significantly lower compared to the case of non-harmonized datasets, with median AUC values close to the chance level. Regarding the classification performance with the internal harmonization approach, we obtain median AUC values ranging between 0.63 and 0.68. These values still show a significant reduction of distinguishability between sites with respect to non-harmonized data. However, in this case, a slight difference between sites is still appreciable. See Additional file 1 for AUC scores for each couple of sites, for the different harmonization approaches, dataset and feature set that we investigated.

### Classification results

**Fig. 3:**
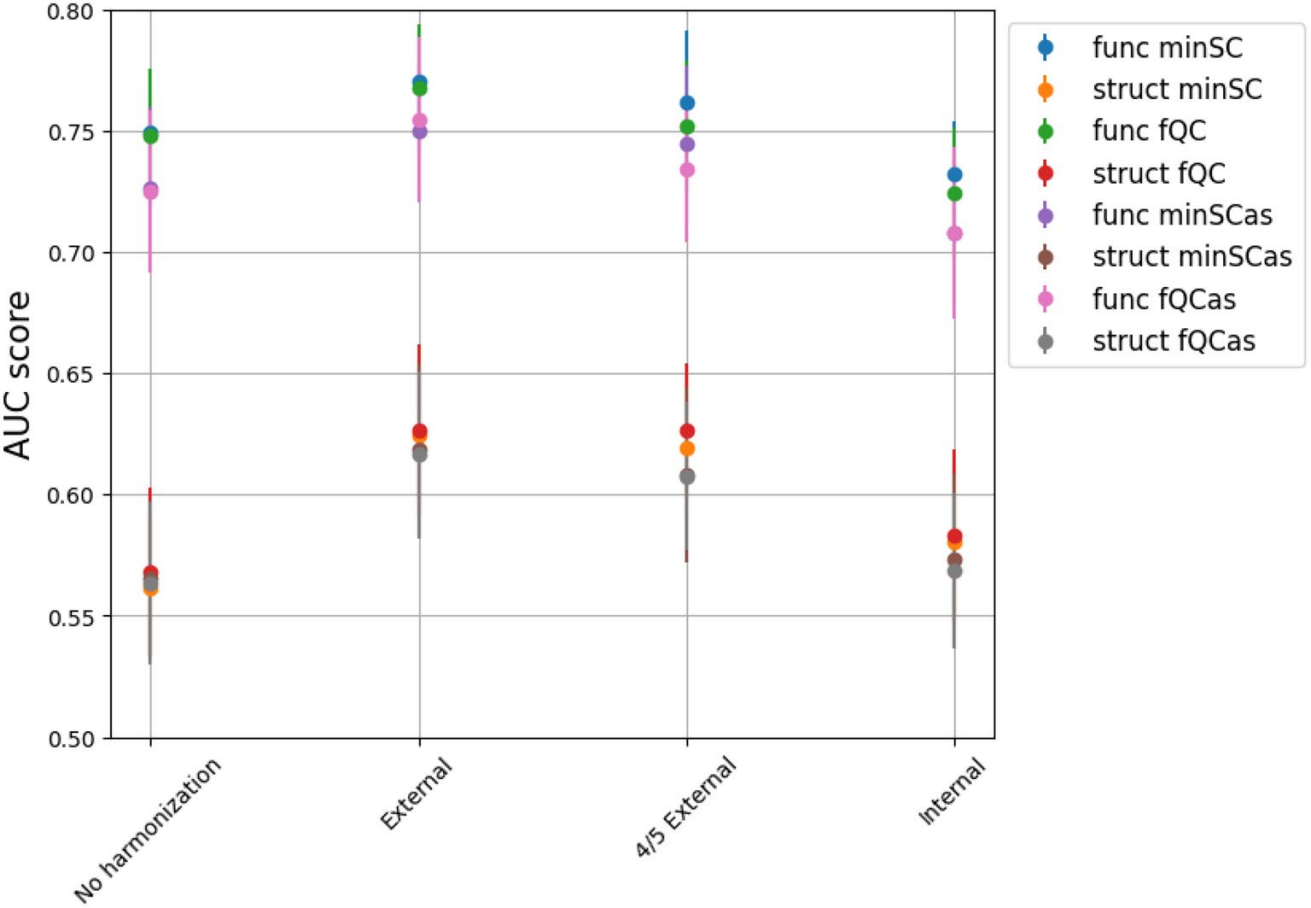
The ASD/TD classification results are reported, for different feature sets and harmonization strategies

The classification results for ASD/TD, using various feature sets, data selections and harmonization strategies, are reported in Fig. 3. The best classification results are obtained for the external harmonization scheme. This result stands for each data selection and for both structural and connectivity features. The 4/5 external harmonization approach shows similar performances. On the other hand, the internal harmonization presents lower performances, comparable (within the fluctuation) with non-harmonized data. The decrease in classification performances for internal harmonization cannot be due to the reduced size of the sample in estimating the harmonization parameters: in fact the dataset used to estimate the model parameters in 4/5 external harmonization has (by design) the same numerosity of the dataset used in internal harmonization.

### Feature importance

**Fig. 4:**
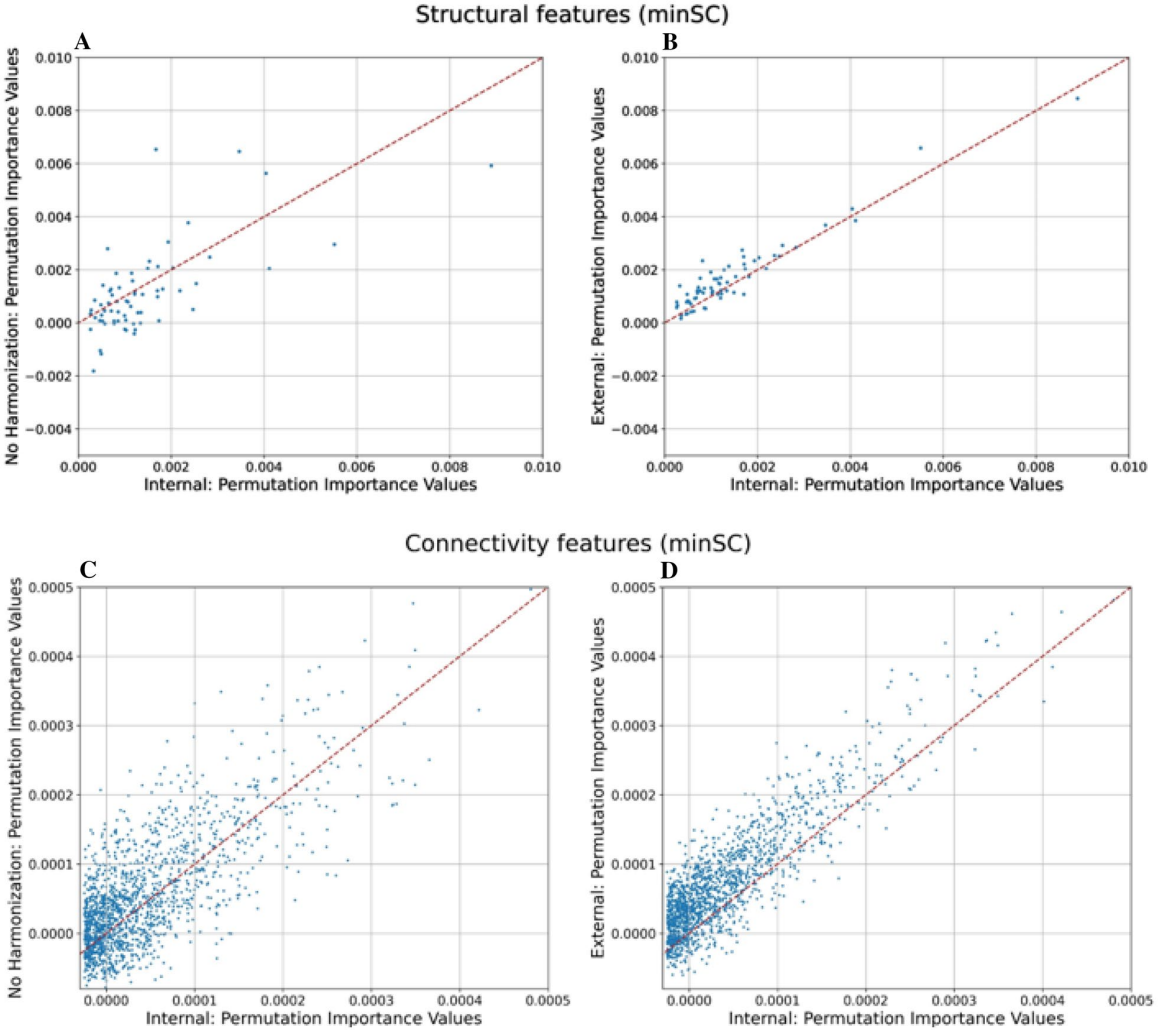
Structural (**A**–**B**) and connectivity (**C**–**D**) features of minSC dataset. Comparison between the no-harmonization and the internal-harmonization criteria (**A**–**C**) and between the external-harmonization and the internal-harmonization criteria (**B**–**D**). We show the 30% of the most important features for the internal-harmonization method in order to see what are the feature importance values of the same features for the no-harmonization method

The importance scores were used to identify the most important features for the SVM model classification for the three harmonization schemes: external, internal and no harmonization. After obtaining the feature permutation importance score for each feature, for both connectivity and structural features, we selected the 30% of the most important features: 1576 for connectivity features and 66 for structural features. In Fig. 4 we show scatter plots of the 30% most important features for no harmonization (left) and for external harmonization (right), versus the ones for internal harmonization. Plots are reported for structural (top) and connectivity (bottom) features, for the minSC dataset. The correlation of the feature importance values of internal harmonization with the feature importance of the other two methods is 67% for plot A in Fig. 4, 96 % for plot B, 74% for plot C, 88% for plot D. The correlation between the three methods is high, especially between the internal and external schemes. For consistency, for a single harmonization scheme, we also checked the correlation among various folds in the cross-validation, which is always in the order of 82% for connectivity features and 96% for structural features (the heatmaps of the inter-method correlations and intra-method correlation between different folds of CV are shown in the Additional file 1).

Then we looked whether there were common important features among the three different harmonization approaches. We found that, in the 1576 most important connectivity features, 1015 (64%) were in common between the three harmonization method using the minSC dataset, and 782 (50%) using the fQC dataset; for the structural features, in the top 66 features, 45 (68%) were in common between the three harmonization methods using the minSC dataset, and 39 (60%) using the fQC dataset. Detailed results are reported in the tables in the Additional file 1: the 30% of the most important features for internal harmonization are presented, with their ranking and importance. The ranking and importance of that features is also reported for the other two methods. Standard deviation of the importance, calculated over the 50 repetitions, is also reported. The common features for the three harmonization methods are marked with “C”, common, while features that are not in the 30% of the most important ones for external harmonization and no harmonization are marked with “NP”, not present.

Correlations seem to show a good agreement of feature importance among different methods. However, by looking at *most important* features, these are generally defined by a threshold: in this case we considered the 30% of the most important features for the internal harmonization. In these cases, a relevant number of these features are absent in the 30% of the most important ones for the other methods, since their importance position changes, showing some differences in the feature importance across the three harmonization methods (these results are shown in the tables in the Additional file 1). This observation leads us to state that the interpretation and evaluation of the most important features, along with their potential implications within a specific pathological context, are influenced by the choice of a specific harmonization approach and dataset.

## Conclusion

Our results show that site distinguishability and classification performances depend on the multicentric data harmonization scheme. The external harmonization, which is the most widely used scheme, presents the best performances in both tasks. These results stand for both structural and connectivity features and for each of the investigated data selections. However, the best performances achieved by the external harmonization scheme do not necessarily imply that this is the correct approach. The use of a cross validation scheme for which the training is not completely blind to the test set, may of course alter these results.

In our opinion, the comparison between the internal harmonization scheme and the 4/5 external one, for which the size of the sample for the estimation of the harmonization parameters is the same, clearly shows the presence of a bias for the second method. Consequently, also the excellent harmonization performances obtained in the external scheme may be affected by this bias. The internal scheme is not able to perform a complete elimination of the site effect, but provides more reliable results due to the complete separation between training and test sets.

The features that most contribute to the classification task differ significantly among the different harmonization schemes. Hence, when a classification task is used to identify brain regions, morphological or connectivity features which are the most involved in the discrimination of a neurological or neurodevelopmental disease, the dependence of these results has to be taken into account. In this case, our suggestion is to use the most rigorous harmonization approach, which is the one carried out only on the training dataset, i.e. inside the CV loop (internal one).

### Supplementary Information

**Additional file 1: Fig. A1.** Site identification for different harmonization strategies for structural features. Left: minSCas data set. Right: fQCas dataset. In order to limit LSS-LES effects, only sites with more than 20 TDs are considered.** Fig. A2.** Site identification for different harmonization strategies for connectivity features. Left: minSCas data set. Right: fQCas dataset. In order to limit LSS-LES effects, only sites with more than 20 TDs are considered.** Fig. A3.** Structural features minSC dataset; Correlation between CV folds, for each harmonization methods (top and bottom-left). Correlation between harmonization methods (bottom-right).** Fig. A4.** Connectivity features minSC dataset; Correlation between CV folds, for each harmonization methods (top and bottom-left). Correlation between harmonization methods (bottom-right).** Fig. A5.** Structural features fQC dataset; Correlation between CV folds, for each harmonization methods (top and bottom-left). Correlation between harmonization methods (bottom-right).** Fig. A6.** Connectivity features fQC dataset; Correlation between CV folds, for each harmonization methods (top and bottom-left). Correlation between harmonization methods (bottom-right)

**Additional file 2.** Sheet TabSSS1 Data selections: Numerosity of each site, for each data selection. Sheet Subjects: Subjects involved in each data selection. Sheet Structural fQC: List of the most important structural features in common for each harmonization method for fQC dataset. Sheet Structural minSC: List of the most important structural features in common for each harmonization method for minSC dataset. Sheet Connectivity fQC: List of the most important connectivity features in common for each harmonization method fQC dataset. Sheet Connectivity minSC: List of the most important connectivity features in common for each harmonization method for minSC dataset.

## Data Availability

I hereby state that the data used can be found in the ABIDE site for download, that in the Additional file 1 we provide the complete list of the subjects used in the data selection for reproducibility and that I can provide all the additional and necessary material to interpret, replicate and build upon the findings reported in the article.

## Abbreviations

AUC: Area under the curve
ABIDE: Autism brain imaging data exchange
ASD: Autism spectrum disorder
C-PAC: Configurable pipeline for the analysis of connectomes
CV: Cross validation
FIQ: Full intelligence quotient
RBF: Gaussian radial basis function
IQR: Interquartile range
LSS-LES: Low sample size/low effect size
ML: Machine learning
MAD: Median absolute deviation
minSC: Minimal selection criteria
minSCas: Minimal selection criteria age selection
PCs: Principal components
ROC: Receiver operating characteristic
fQC: Selection criteria based on fMRI quality controls
fQCas: Selection criteria based on fMRI quality controls age selection
SVC: Support vector classifier
SVM: Support vector cachines
TD: Typically developing
