# Supplementary material for: Effect of data harmonization of multicentric dataset in ASD/TD classification
Source: Brain Inform. 2023 Nov 25;10(1):32. doi: 10.1186/s40708-023-00210-x (PMC10676338; doi:10.1186/s40708-023-00210-x)
Supplement: Supplementary file 1 — Additional file 1: Fig. A1. Site identification for different harmonization strategies for structural features. Left: minSCas data set. Right: fQCas dataset. In order to limit LSS-LES effects, only sites with more than 20 TDs are considered. Fig. A2. Site identification for different harmonization strategies for connectivity features. Left: minSCas data set. Right: fQCas dataset. In order to limit LSS-LES effects, only sites with more than 20 TDs are considered. Fig. A3. Structural features minSC dataset; Correlation between CV folds, for each harmonization methods (top and bottom-left). Correlation between harmonization methods (bottom-right). Fig. A4. Connectivity features minSC dataset; Correlation between CV folds, for each harmonization methods (top and bottom-left). Correlation between harmonization methods (bottom-right). Fig. A5. Structural features fQC dataset; Correlation between CV folds, for each harmonization methods (top and bottom-left). Correlation between harmonization methods (bottom-right). Fig. A6. Connectivity features fQC dataset; Correlation between CV folds, for each harmonization methods (top and bottom-left). Correlation between harmonization methods (bottom-right) [file 40708_2023_210_MOESM1_ESM.docx]

Tables

In file Supplementary Tables:

- Numerosity of each site, for each data selection
- Subjects involved in each data selection
- List of the most important structural features in common for each harmonization method and for both, fQC and minSC, datasets.
- List of the most important connectivity features in common for each harmonization method and for both, fQC and minSC, datasets.

Figures


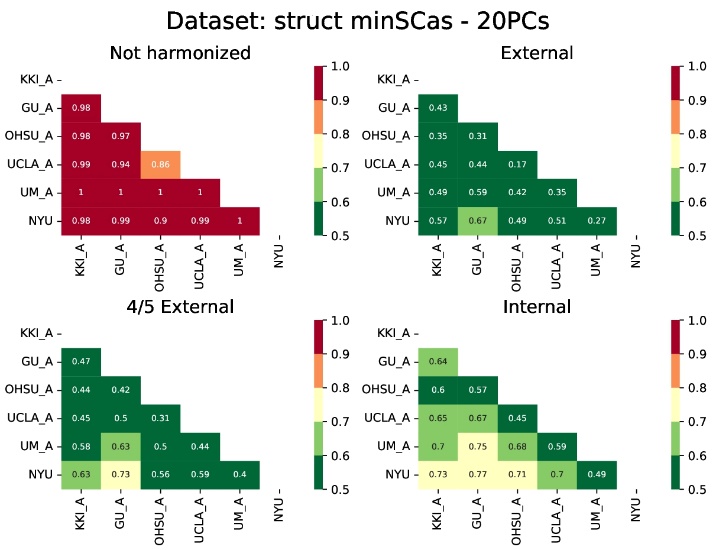

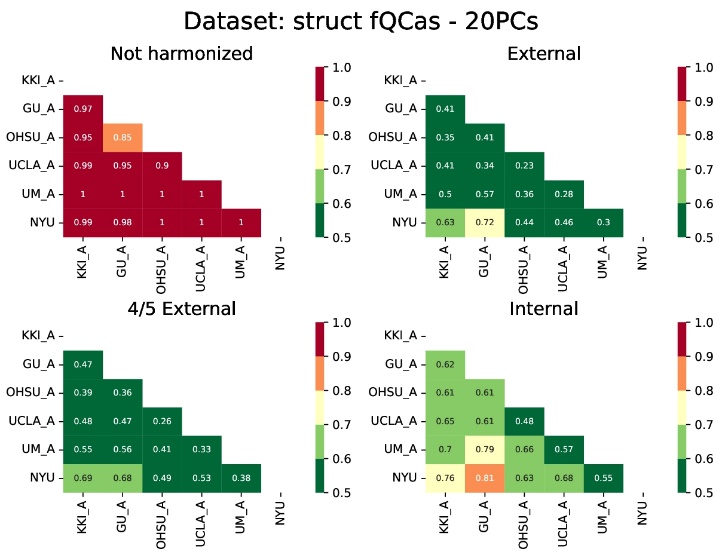


Fig.A1: Site identification for different harmonization strategies for structural features. Left: minSCas data set. Right: fQCas dataset. In order to limit LSS-LES effects, only sites with more than 20 TDs are considered.


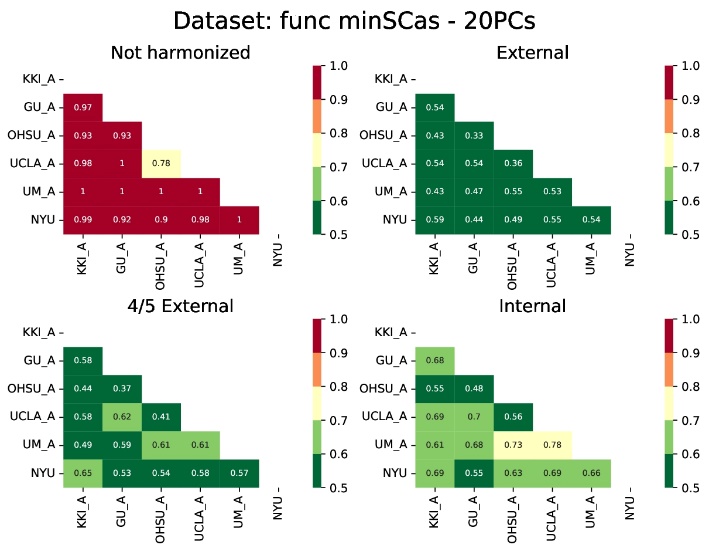

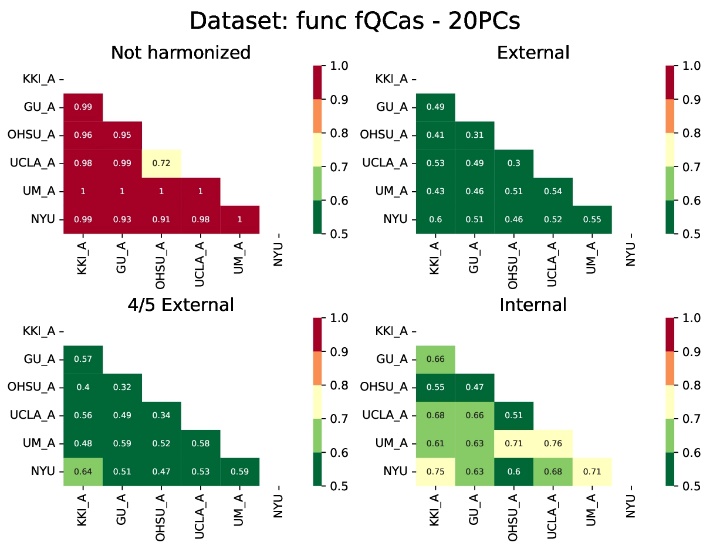


Fig.A2: Site identification for different harmonization strategies for connectivity features. Left: minSCas data set. Right: fQCas dataset. In order to limit LSS-LES effects, only sites with more than 20 TDs are considered.


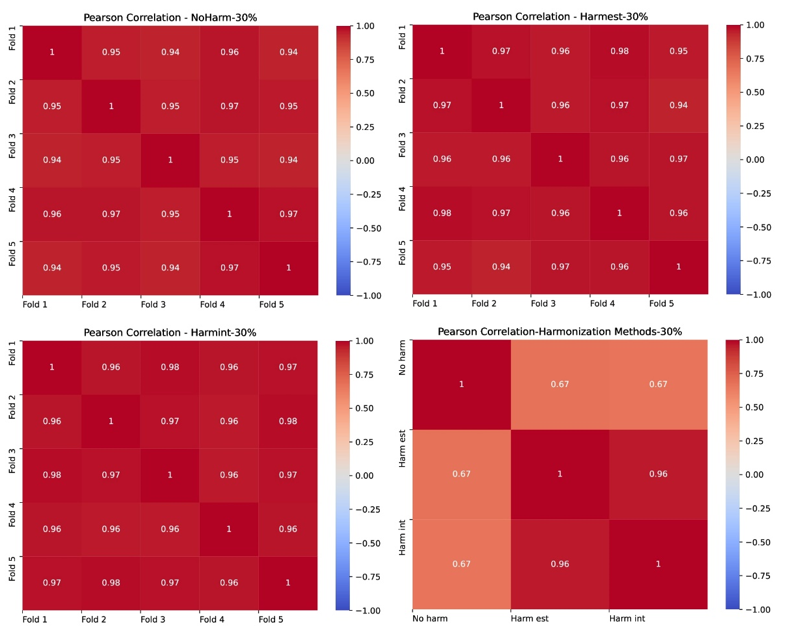


Fig. A3: Structural features minSC dataset; Correlation between CV folds, for each harmonization methods (top and bottom-left). Correlation between harmonization methods (bottom-right).


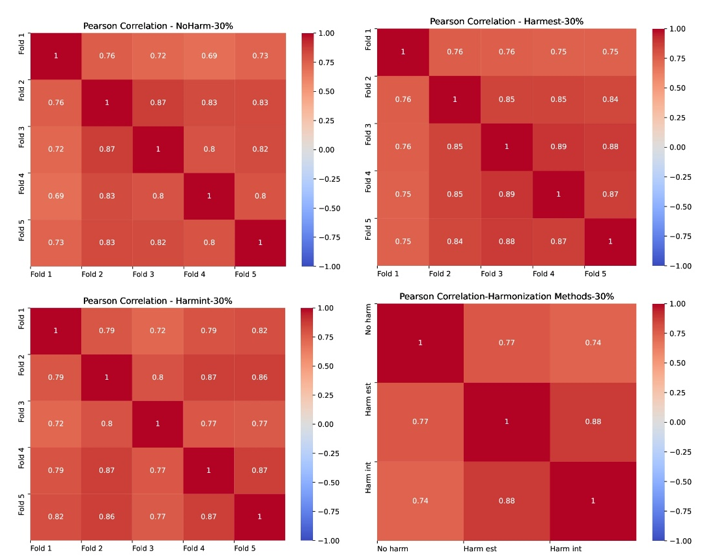


Fig. A4: Connectivity features minSC dataset; Correlation between CV folds, for each harmonization methods (top and bottom-left). Correlation between harmonization methods (bottom-right).


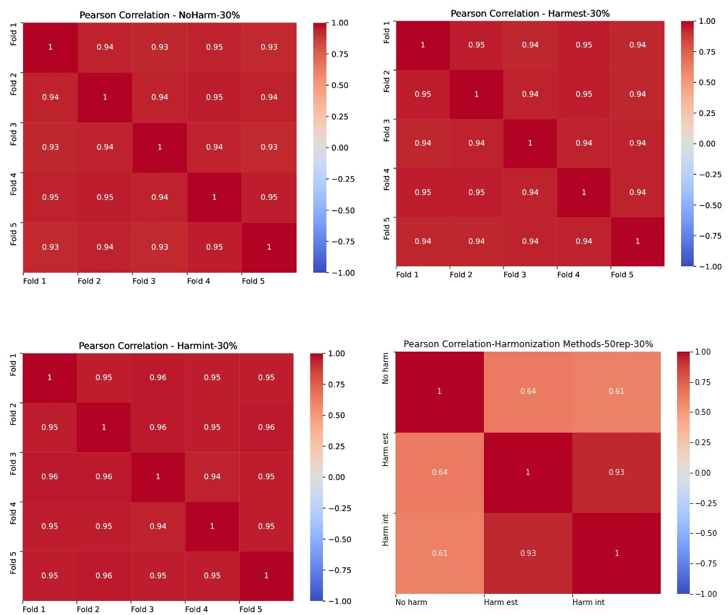


Fig. A5: Structural features fQC dataset; Correlation between CV folds, for each harmonization methods (top and bottom-left). Correlation between harmonization methods (bottom-right).


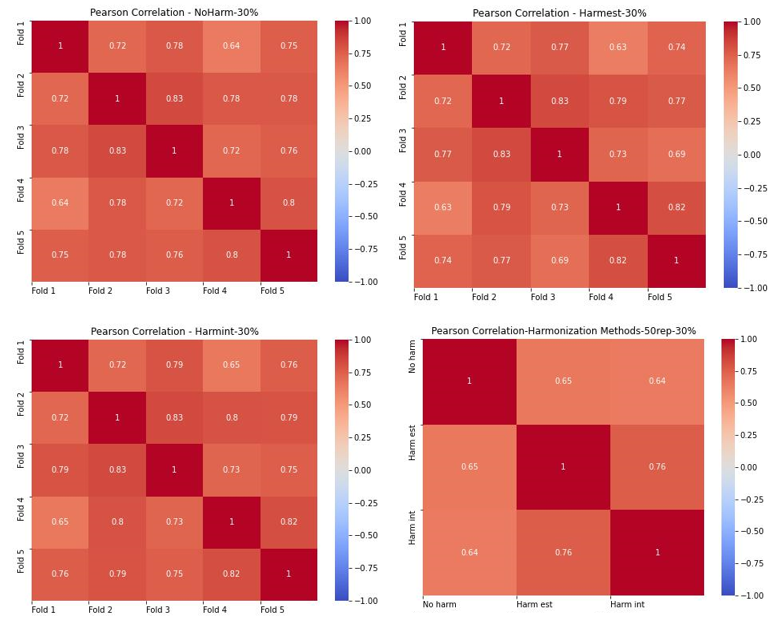


Fig. A6: Connectivity features fQC dataset; Correlation between CV folds, for each harmonization methods (top and bottom-left). Correlation between harmonization methods (bottom-right).
